# Infant’s Behaviour Checklist for low birth weight infants and later neurodevelopmental outcome

**DOI:** 10.1038/s41598-021-98884-y

**Published:** 2021-09-29

**Authors:** Hideki Kihara, Hisako Nakano, Tomohiko Nakamura, Hirotaka Gima

**Affiliations:** 1 Babycastle Corporation, 373-1, Tsubuku-imamachi, Kurume City, Fukuoka 830-8630 Japan; 2 Department of Physical Therapy, Kyorin University, 5-4-1 Shimorenjaku, Mitaka City, , Tokyo 181-8612 Japan; 3 Department of Neonatology, Nagano Children’s Hospital, 3100, Toyoshina, Azumino City, Nagano 399-8288 Japan; 4 Department of Physical Therapy, Faculty of Health Sciences, Tokyo Metropolitan University, 7-2-10 Higashi-Ogu, Arakawa-ku, Tokyo, 116-8551 Japan; 5 Department of Physical Therapy, Graduate School of Human Health Sciences, Tokyo Metropolitan University, 7-2-10 Higashi-Ogu, Arakawa-ku, Tokyo, 116-8551 Japan

**Keywords:** Paediatric research, Autism spectrum disorders

## Abstract

Assessment of the characteristics of spontaneous movements and behaviour in early infancy helps in estimating developmental outcomes. We introduced the Infant Behaviour Checklist (IBC) and examined the relationship between the behavioural characteristics of low-birth-weight infants and neurodevelopmental outcomes at 6 years of age. The behavioural characteristics during the neonatal (36–43 weeks, adjusted) and early infancy periods (49–60 weeks, adjusted) were assessed in very-low-birth-weight infants. The IBC includes 44 common behaviours. We assessed the appearance of individual behavioural characteristics at each period according to the neurodevelopmental outcome. Of the 143 infants assessed during the neonatal period, 89 had typical development (TD), 30 had intellectual disability (ID), and 24 had autism spectrum disorder (ASD). In 78 infants assessed during early infancy, 40, 21, and 17 had TD, ID, and ASD, respectively. The frequency of appearance of three behaviour-related items was significantly lower in the ID group than in the TD group. The frequency of appearance of three posture- and behaviour-related items was significantly lower, while that of two posture-related items was significantly higher, in the ASD group than in the TD group. Behavioural assessment using the IBC may provide promising clues when considering early intervention for low-birth-weight infants.

## Introduction

Developments in medical technology have markedly improved the survival rates of low-birth-weight infants; however, such infants have increased risks of neurodevelopmental abnormalities^1,2^. Several studies have also reported that preterm birth and low-birth-weight are risk factors for future developmental disabilities such as intellectual disability (ID)^3,4^ and autism spectrum disorder (ASD)^5–9^. In recent years, assessing neurological development based on the characteristics of spontaneous movements and behaviours can be useful in considering interventions for infants at risk of developmental problems. In particular, it has been reported that a type of spontaneous movement, defined by Prechtl as general movements (GMs)^10^, is an early sensitive marker of cerebral palsy (CP) later in life^11,12^. Furthermore, a relationship between the qualitative abnormality of GMs, ID^13,14^, and ASD^15–17^ has been reported, but its accuracy is still inadequate compared with that of the relationship with CP. Regarding ID and ASD, observing the various behavioural characteristics rather than the qualitative abnormalities of GMs may be useful as an early sign^18–26^.

We have focused on the characteristics of spontaneous movements in early infancy and reported their relationship with neurological development^26–29^. We have reported that it is useful to quantify the various spontaneous movements that infants show; however, a detailed evaluation of their behavioural characteristics has thus far been sufficient. Therefore, we conducted a detailed observational evaluation of behavioural characteristics during early infancy in low-birth-weight infants using videos recorded for the assessment of GMs. In this study, we introduced the Infant’s Behaviour Checklist (IBC) and examined the relationship between behavioural characteristics of low-birth-weight infants assessed using the IBC and their neurodevelopmental outcomes at 6 years of age. The IBC lists 44 items considered important in assessing spontaneous movement and behavioural and postural characteristics of low-birth-weight infants. Clarifying the relationship between detailed behavioural characteristics in early infancy and later neurological development is important for enhancing developmental care for low-birth-weight infants.

## Results

### Participant characteristics

The distribution of the developmental outcomes at age 6 years in neonatal assessment (NA) was as follows: typical development (TD), n = 89; ID, n = 30; and ASD, n = 24. The distribution of the developmental outcomes at age 6 years in early infancy assessment (EA) was as follows: TD, n = 40; ID, n = 21; and ASD, n = 17. For the 45 infants who belonged to both groups, the distribution of the developmental outcomes at age 6 years was as follows: TD, n = 25; ID, n = 11; and ASD, n = 9.

The background factors of NA and EA are listed in Tables 1 and 2, respectively. In the NA group, the birth weight was significantly lower in the ASD than in the TD group. In the EA group, there were no significant differences between the groups in terms of birth weight, gestational age (weeks), or age in weeks at video recording.

**Table 1:** Characteristics and developmental outcome of the neonatal assessment group.

	TD	ID	ASD
Number	89	30	24
Male/female (n)	30/59	17/12	14/10
Birthweight (g), median (range)	1,058 (489–1495)	931 (460–1498)	827* (588–1346)
Gestational age (weeks and days), median (range)	28w5d (22w5d–34w4d)	27w2d (23w0d–36w3d)	27w2d (23w6d–33w0d)
Age at recording of behavioural development (weeks and days), median (range)	38w0d (36w0d–43w2d)	39w2d (36w1d–43w4d)	39w5d (36w4d–43w1d)

*TD* typical development, *ID* intellectual disability, *ASD* autism spectrum disorder.

*p < 0.05 in comparison with TD group using the Mann–Whitney U test.

**Table 2:** Characteristics and developmental outcome of the early infancy assessment group.

	TD	ID	ASD
Number	40	21	17
Male/female (n)	13/27	7/14	9/8
Birthweight (g), median (range)	983 (492–1468)	790 (502–1498)	849 (546–1414)
Gestational age (weeks and days), median (range)	28w6d (23w2d–32w4d)	27w0d (23w0d–36w3d)	27w2d (23w6d–33w0d)
Age at recording of behavioural development (weeks and days), median (range)	54w0d (49w0d–58w1d)	53w1d (49w6d–59w0d)	53w3d (50w0d–60w2d)

*TD* typical development, *ID* intellectual disability, *ASD* autism spectrum disorder.

*p < 0.05 in comparison with TD group using the Mann–Whitney U test.

### Evaluation of the behavioural characteristics using IBC

The frequency of observation of each behaviour in all groups for NA and EA is shown in Table 3. In the NA group, the ID group exhibited significantly fewer instances of trunk rotation (item 19, 10%), separate finger flexion and extension (item 25, 20%), and lower limb flexion and hip internal rotation and external rotation (item 27, 27%) than did the TD group. In contrast, there were no items in the ASD group that showed a significant difference. In the EA group, the ID group showed significantly fewer instances of toe flexion and extension separately (item 32, 19%) than did the TD group. Meanwhile, the ASD group showed significantly more instances of asymmetrical tonic neck reflex (ATNR) (item 3, 35%) and toe flexion (item 16, 41%), and there were significantly fewer instances of head at the midline (item 2, 0%), neck rotation (item 18, 29%), and wrist rotation (item 24, 0%).

**Table 3:** Percentage of presence behaviour in all items by group in the neonatal and early infancy assessment.

Item number	Neonatal assessment group			Early infancy assessment group		
	TD (n = 89)	ID (n = 30)	ASD (n = 24)	TD (n = 40)	ID (n = 21)	ASD (n = 17)
1	2	3	4	8	19	18
2	–	–	–	33	10	0*
3	3	0	4	5	14	35*
4	1	0	4	0	5	6
5	1	7	0	0	10	0
6	39	60	50	28	24	35
7	18	10	13	50	43	24
8	6	10	4	5	10	6
9	20	23	50	3	0	24
10	7	7	4	8	0	0
11	6	3	4	3	0	0
12	20	17	13	20	10	0
13	–	–	–	78	62	59
14	10	10	13	0	0	0
15	7	13	17	3	5	6
16	–	–	–	8	24	41*
17	10	10	8	8	0	0
18	30	10	30	75	43	29*
19	44	10*	21	25	24	18
20	6	10	4	0	0	6
21	76	80	54	73	52	47
22	18	7	4	65	43	47
23	7	13	4	0	5	0
24	–	–	–	43	19	0*
25	53	20*	17	38	29	35
26	3	13	8	10	14	6
27	56	27*	33	58	29	53
28	1	3	4	18	0	0
29	45	27	29	93	86	82
30	16	0	17	40	19	12
31	–	–	–	98	81	100
32	–	–	–	63	19*	41
33	8	0	8	25	24	24
34	53	27	29	35	19	41
35	38	23	8	18	14	12
36	55	43	33	5	10	12
37	10	7	0	10	5	6
38	–	–	–	3	5	6
39	49	27	25	53	43	53
40	74	67	79	0	0	12
41	45	57	54	0	0	6
42	8	17	13	8	0	6
43	5	17	17	0	0	0
44	1	0	8	0	0	0

*TD* typical development, *ID* intellectual disability, *ASD* autism spectrum disorder.

*p < 0.05 in comparison with TD group using the Fisher’s exact test (corrected by the Benjamini–Hochberg method).

**Figure 1:**
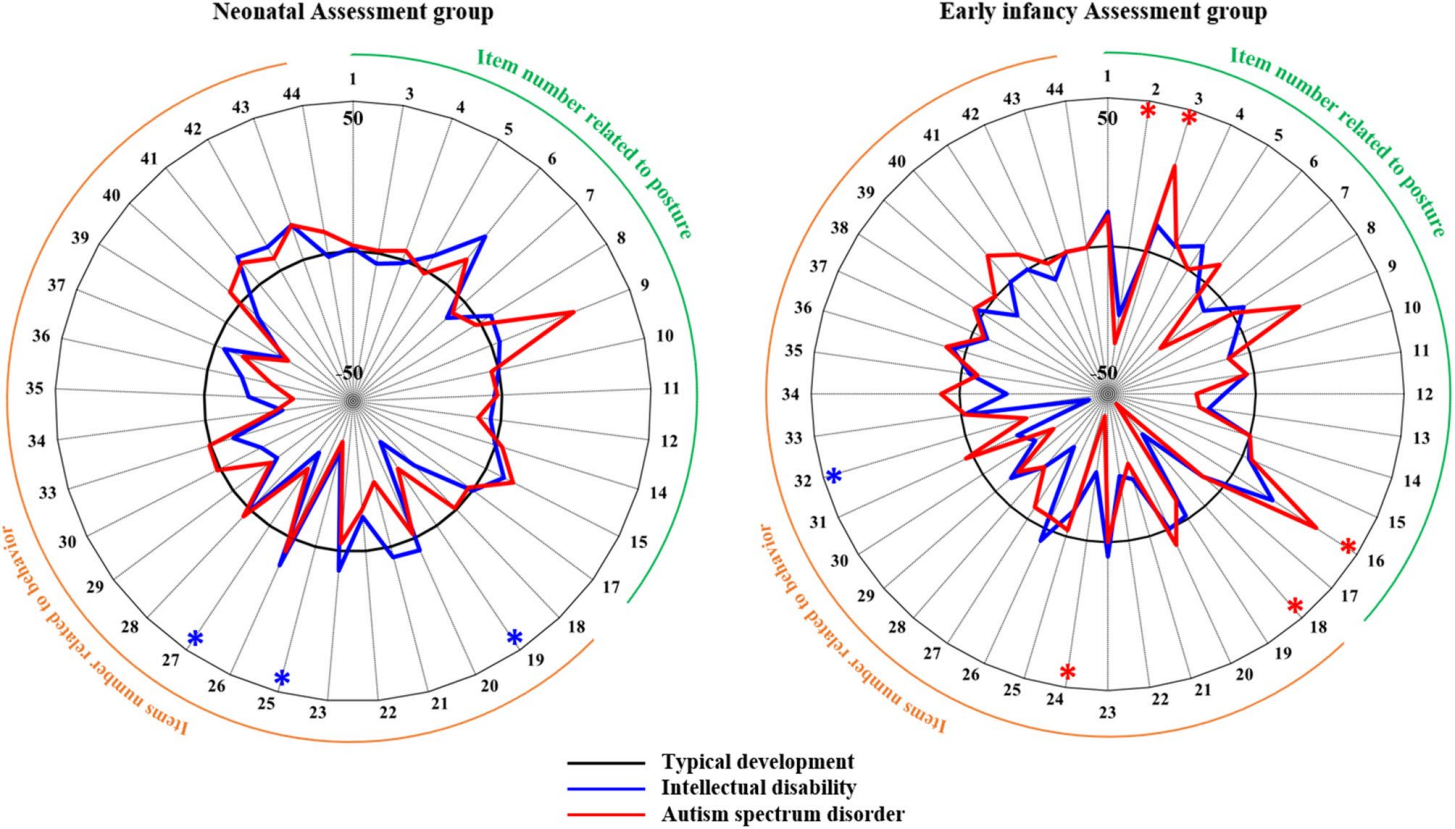
Radar chart of the difference percentage between the typical development group and the other group at NA and EA terms. The red and blue line shows the distribution of the difference of percentage of participants for whom the posture and behaviour appeared. Each line represents the value of the TD group minus the value of the ID and the ASD group (note that the values of the TD group are all zero and the values have no units). *p < 0.05 in comparison with the TD group using Fisher’s exact test corrected by the Benjamini–Hochberg method.

Figure 1 shows the distribution of the data for each item in the ID and the ASD groups at NA and EA terms by the difference of percentage of participants for whom the posture and behaviour appeared. Each line represents the value of the TD group minus the value of the ID (blue line) and the ASD (red line) group, with larger positive values indicating a higher proportion of appearance compared to the TD group, and larger negative values indicating a lower proportion of appearance.

## Discussion

In this study, we introduced the IBC and examined the relationship between the behavioural characteristics of low-birth-weight infants assessed using IBC and their neurodevelopmental outcomes at 6 years of age. In particular, we focused on ID and ASD as developmental outcomes, and examined the characteristics of spontaneous movements and behaviour during the neonatal and early infancy periods. These periods are considered to be important for qualitative evaluation of the characteristics of spontaneous movements^10–12,30^ and also from the viewpoint of the developmental mechanism of the central nervous system^10,31–33^. In particular, fidgety movements (FMs) which appear during the EA period, have been reported to be strongly related to later neurological developmental outcomes^10–17,29–33^. The assessment of characteristics of spontaneous movements and behaviour at both NA and EA is considered important to verify early motor signs of ID and ASD.

Evaluation using the IBC revealed the following behavioural characteristics in the ID group: (1) Among the items of IBC, a significantly lower frequency of appearance was found in some items related to behaviour, but not in items related to posture. (2) The number of items related to behaviour with a significantly lower frequency of appearance was larger in the NA group than that in the EA group. In the ID group, the frequencies of items 19 (trunk rotation), 25 (separate finger flexion and extension), and 27 (lower limb flexion and hip internal rotation and external rotation) were significantly lower in NA; in contrast, the frequency of item 32 (toe flexion and extension separately) was significantly lower in EA. Meanwhile, there were no items related to posture that showed a significant difference between NA and EA. The characteristic behaviour in the ID group may reflect the low quantitative characteristics of spontaneous movements and behaviour in the ID group. A previous study that examined the characteristics of spontaneous limb movements by computer-based analysis using the same image data as in this study reported that the value of the quantitative indices (average velocity and number of movement units per min) in the NA was significantly lower in the ID than in the TD group^27^. In the evaluation using the IBC, items related to behaviour were judged ‘present’ when the behaviour was observed at least twice. Since a reduction in the activity of spontaneous movements also leads to a reduced frequency of appearance of each behaviour, it is considered that a significantly lower frequency of appearance in items related to behaviour was observed in the ID group. However, a previous study examining the characteristics of spontaneous limb movement in EA reported no significant difference in the values of quantitative indices between the ID group and the TD group^26^. The quantitative characteristics of spontaneous movements in the ID group may be more apparent in the NA than in the EA group. The results of the present study also indicated that the quantitative characteristics of spontaneous movements and behaviour in the ID group may appear more prominently in the NA than in the EA group, suggesting the importance of evaluating NA for the ID group.

The important results in the evaluation of the ASD group were as follows: (1) There were no items in the NA group that showed a significant difference. (2) In the EA group, there were many items related to posture that showed a significant difference in the appearance rate. (3) All items that showed a significant difference in the appearance rate were related to the movement of the neck and distal part of the upper and lower limbs. Previous studies that examined early signs of ASD by assessing the characteristics of spontaneous movements, posture, and behaviour reported features, such as abnormal upper extremity muscle tone^21^, postural asymmetries during lying^18,20^, head-lag during the pull-to-sit transition^23^, oral-motor abnormalities^22^, motor developmental delay in early infancy^24^, qualitative abnormalities in GMs^16,17^, poorer performance in maintaining the midline position of the head^26^, and lower motion complexity in legs^34^. All these early signs of ASD have been observed after 3 months of age. Therefore, it is highly possible that the results of this study also contain more information on the early behavioural characteristics of ASD in EA than in NA. In the EA evaluation, the frequencies of items 2 (head midline), 18 (neck rotation), and 24 (wrist rotation) were significantly lower, and 3 (ATNR) and 16 (toe flexion) were significantly higher in the ASD than in the TD group. Of these, items related to head movement and position (head midline, neck rotation, and ATNR) were considered to reflect the poorer performance in maintaining the midline position of the head in the previous study^26^. Meanwhile, the small amount of wrist rotation and the high ratio toe flexion may reflect the abnormality of FMs in ASD. FMs are a type of GM characterized by small continual movements of moderate speed and variable acceleration of the neck, trunk, and limbs in all directions that appears in early infancy and is defined as ‘an ongoing stream of small and circular movements of limbs’^32,33^. A study examining the kinematic features of FMs reported that the value of curvature in the movement trajectory of the distal limbs was related to the frequency of FMs appearance^35^. This value of the curvature is the magnitude by which a trajectory deviates from being straight and reflects the fine rotational movement of the wrist and the ankle. Previous studies have reported that the appearance of FMs is often absent in infants with ASD^15–17^, and the characteristics of distal limb movements and posture in the ASD group in this study were considered to be related to FMs abnormalities (absence of FMs), similar to results of previous studies.

Preterm birth and low-birth-weight are risk factors for ASD^5–9^. However, detecting the early motor signs of ASD during early infancy is a considerable challenge. We used the IBC to investigate the relationship between behaviours in the neonatal and early infancy periods with development at the age of 6 years due to disability. We also ascertained the characteristics of behaviours by disability in the neonatal and early infancy periods. Behavioural characteristics during early infancy in low-birth-weight infants reflected the diagnosis of later ID and ASD. Identifying factors that allow the early recognition of ASD is crucial because early intervention results in the development of better socially engaged imitation, eye contact, cognitive ability, adaptive behaviour, socialization, daily living skills, and motor skills for ASD^36,37^. Behavioural assessment using the IBC may provide promising clues when considering early intervention for low-birth-weight infants.

The limitations of this study need to be considered. Firstly, for consistency, the observational assessments were all performed by one assessor. In clinical practice, it would be important to have more than three assessors to ensure a reliable evaluation. Secondly, our study’s assessments using the IBC did not compare the severity of disabilities or scoring. Furthermore, several participants (13 of 24 in the NA group and 3 of 17 in the EA group) in the ASD group had difficulty in completing the Wechsler Intelligence Scale for Children (WISC) III due to the characteristics of ASD; thus, their intelligence quotient (IQ) could not be assessed accurately (see Supplementary Fig. S1 online). This is an important issue worthy of future investigation. Therefore, we would like to conduct further studies using a larger sample size to investigate the disability severity grading and scoring and the impact of comorbid ASD and ID.

## Methods

### Ethics

The present study was approved by the Institutional Review Board of Nagano Children’s Hospital. The infants’ parents or guardians were provided with both written and oral explanations of how the data would be handled (use in written reports and presentations at academic conferences, protection of private information) before they provided signed consent. All procedures were performed in accordance with the approved guidelines and regulations.

### Participants

**Figure 2:**
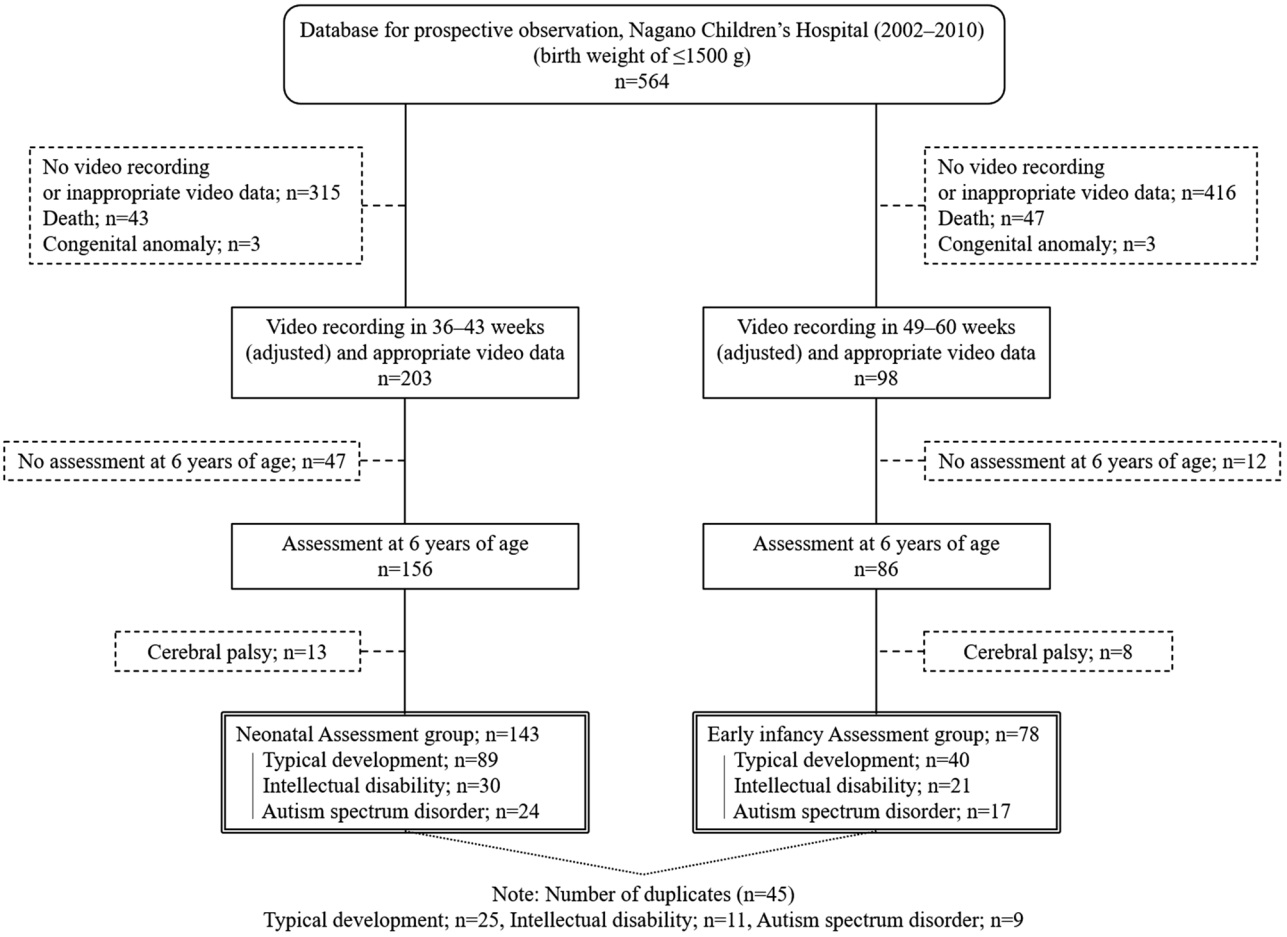
Flow diagram showing study participant enrolment for behavioural assessment during the neonatal and early infancy periods.

We included 564 extremely-low-birth-weight infants who were hospitalized in the neonatal intensive care unit of Nagano Children’s Hospital between 2002 and 2010. We assessed the behaviours of 143 such infants during the neonatal period (36 weeks 0 days to 43 weeks 6 days, adjusted, the NA group) and 78 infants in the early infancy period (49 weeks 0 days to 60 weeks 6 days, adjusted, the EA group) (Fig. 2).

### Recording of behaviour

The infants’ behaviours were recorded by videography (DCR-TRV20 or DVR-PC350, SONY, Tokyo, Japan). The video recording method was modelled on that of Einspieler et al.^23^ The infants in the NA group were placed supine on a cot in the neonatal intensive care unit, and those in the EA group were placed supine on an outpatient bed mat wearing light clothing and diapers. The infants were monitored for 30 min. Video recordings were made at 3–5-min intervals, during which they were spontaneously moving without being excited or crying. Video recordings were recorded 1 h before or after nursing. The video recordings were made with a camera placed on a tripod directly above the infants. Using the videos, we assessed the behaviour over approximately 1 min during which the infants were alert and awake and showed continuous movements.

### Assessment of behaviour in IBC

The behaviours and assessment criteria for the IBC are listed in Table 4. In this study, we have developed a list of observation items based on methods from the Neurological Assessment of the Preterm and Full-term Newborn Infant^38^ (item numbers 9, 16, 17, and 40–44) and the Newborn Individualized Developmental Care and Assessment Program^39^ (item numbers 33–39), which are commonly used in clinical practice to evaluate the behaviour of infants. In addition, the other items were picked up for postures and behaviour that the authors feel were commonly observed in clinical practice.

**Table 4:** Items of Infant’s Behaviour Checklist (IBC) and assessment criteria.

	No	Item	Assessment criteria
Items related posture	1	Neck extension	Maintains neck extension (at least 10°); neck at the midline or rotated
	2*	Head at the midline	Maintains head at the midline; neck at the midline
	3	Asymmetrical tonic neck reflex	Asymmetric tonic neck reflex; upper and lower limbs extended toward the face, flexed on the opposite side
	4	Trunk extension	Maintains trunk extension (at least 10°); trunk at the midline or rotated
	5	Body co-contraction	Overexerted body; limb and trunk muscles contracted simultaneously
	6	Elbow flexion	Elbows maintained in the flexed position (at least 90°); shoulder rotated inward
	7	Elbow extension	Elbows maintained in the extended position (less than 10°); shoulders rotated inward
	8	Finger semi-flexion	Fingers incompletely flexed and not grasping
	9	Thumb adduction	Thumbs maintained in the adducted position toward the palm (at least 60°); other fingers extended
	10	Lower limb crossing	Hips maintained in the adducted position (at least 10°); hips in flexed position
	11	Lower limb abduction	Hips maintained in the abducted position (at least 10°); hips in flexed position
	12	Lower limb flexion and elevation	Hips and knees maintained in the flexed position (both at least 60°); feet off the floor
	13*	Lower limb extension and elevation	Maintain the hips in flexed position (at least 60°) and the knees in extended position (less than 10°); feet off the floor
	14	Lower limb extension and ankle dorsiflexion	Maintain the hips and knees extended and ankles in the dorsiflexed position (at least 10°)
	15	Lower limb extension and ankle plantar flexion	Maintain the hips and knees extended and ankles in the plantar flexed position (at least 10°)
	16*	Toe flexion	Five toes maintained in the flexed position
	17	Great toe extension	Great toe maintained in the extended position (at least 10°); other toes at the midline or flexed
Items related behaviour	18	Neck rotation	Neck rotated (at least 10°); rotation crosses the midline
	19	Trunk rotation	Trunk rotated (at least 10°); trunk flexed or extended
	20	Trunk lateral bending	Trunk bent laterally (at least 10°); trunk in the flexed or extended position
	21	Upper limb flexion and forearm pronation and supination	Elbow flexed (at least 60°) and forearm rotated inward or outward (both at least 10°)
	22	Upper limb extension and forearm pronation and supination	Elbow extended (less than 10°) and forearm rotated inward or outward (both at least 10°)
	23	Upper limb swing	Repeated swinging of the upper limb; repeated raising and lowering with the upper limb extended
	24*	Wrist rotation	Wrist rotated; hand movement in the following order: palmar flexion, radial flexion, dorsal flexion, and ulnar flexion
	25	Separate finger flexion and extension	Five fingers of the hand flexed and extended separately
	26	Kicking	Lower limbs repeatedly flexed and extended; lower limbs flexed in turn
	27	Lower limb flexion and hip internal rotation and external rotation	Repetitive internal and external rotation of the hips (at least 10°) with hips and knees flexed (both at least 60°)
	28	Lower limb extension and hip internal rotation and external rotation dorsiflexion	Repetitive internal and external rotation of the hips (at least 10°) with hips and knees extended (both less than 10°)
	29	Lower limb flexion and ankle dorsiflexion and plantar flexion	Repetitive dorsiflexion and plantarflexion of ankles (at least 10°) with hips and knees flexed (both at least 60°)
	30	Lower limb extension and ankle dorsiflexion and plantar flexion	Repetitive dorsiflexion and plantarflexion of ankles (at least 10°) with hips and knees extended (both less than 10°)
	31*	Ankle inversion and eversion	Repetitive inversion and eversion of ankles
	32*	Toe flexion and extension separately	Five toes separately flexed and extended
	33	Kicking the floor	In the supine position; knees flexed and the plantar surface of the feet kicking the floor
	34	Hand to head and ear movement	Hand touching the head or the ear; fingers or palm in contact
	35	Hand to face movement	Hand touching the face; fingers or palm in contact
	36	Hand to mouth movement	Hand touching the mouth; fingers or palm in contact
	37	Hand to hand movement	Both hands touching; fingers or palms in contact
	38*	Hand to knee movement	Hand touching the knee; fingers or palm in contact
	39	Foot to leg movement	Foot touching the other lower limb; toes or the plantar surface of the foot in contact
	40	Single limb tremor	Trembling of one limb
	41	Two or more limb tremor	Trembling of at least two limbs; simultaneous tremors
	42	Startle response	Spontaneous Moro reflex; all four limbs simultaneously extended and outward
	43	Repeat tremor and startle response	Repeated tremors and startled response within 10 s
	44	Tremor and startle response with a cry	Tremor and startle response accompanied by crying

*Assessed only during the early infancy period.

Items related to posture (item numbers 1 − 17) were judged ‘present’ when the condition was observed for more than half of the observation time; if not, they were judged ‘absent’. This criterion is based on the criteria for evaluating the temporal organization of fidgety movements in the Assessment of General Movements^30^ and the activity in the Neonatal Behavioral Assessment Scale^40^.

Items related to behaviour (item numbers 18 − 44) were judged ‘present’ when the behaviour was observed at least twice (uni- or bilaterally); if not, they were judged ‘absent’. Seven of the 44 items (item numbers 2, 13, 16, 24, 31, 32, and 38) were not included in the NA assessments because neonates cannot hold their heads upright or coordinate separate peripheral movements. For consistency, assessments using the IBC were performed by a single physical therapist (H.K.). Note that the assessor was blinded with regards to the participants’ characteristics (sex, birth weight, gestational age, and developmental outcome).

### Assessment of developmental outcome at 6 years of age

Developmental outcomes were determined for children aged 6 years. The WISC III was administered at the development outpatient department of Nagano Children’s Hospital. The tests were administered by a speech-language-hearing therapist or a clinical psychologist. The WISC is an analytical intelligence test applied to children aged 5 to 16 years, in which verbal IQ, performance IQ, and full-scale IQ are each assessed as a deviation IQ (mean 100, one standard deviation 15) indicating their relative position in the same age group^41,42^. In this study, according to the criteria of the WISC, full-scale IQ of ≥ 80 was considered normal; 70–79, borderline; and ≤ 69, delayed. Diagnoses were made by a paediatric psychiatrist using the results of the WISC, brain imaging findings, and the criteria listed in the Diagnostic and Statistical Manual of Mental Disorders (fourth edition). Based on the diagnoses, the children were assigned to the TD, ID, ASD, or CP groups. Children who could not be diagnosed with ASD or CP but who had an IQ of ≥ 80 were placed in the TD group, and those with an IQ of ≤ 79 were placed in the ID group.

### Statistical analyses

The background characteristics of all groups were compared using the Mann–Whitney U test. We calculated the frequency of observance of each behaviour in each group. Fisher’s exact test was used to compare the frequency of behaviours in the ID, ASD, and TD groups. Note that we corrected the p-values using the Benjamini–Hochberg method to account for the effect of false positives due to multiple testing^43^. The False Discovery Rate was set to 0.05 in order to carry out an exploratory test to find the important items.

## Supplementary Information

Supplementary Figure S1.
