# Supplementary material for: Infant’s Behaviour Checklist for low birth weight infants and later neurodevelopmental outcome
Source: Sci Rep. 2021 Sep 29;11:19286. doi: 10.1038/s41598-021-98884-y (PMC8481230; doi:10.1038/s41598-021-98884-y)
Supplement: Supplementary file 1 — Supplementary Figure S1. [file 41598_2021_98884_MOESM1_ESM.docx]

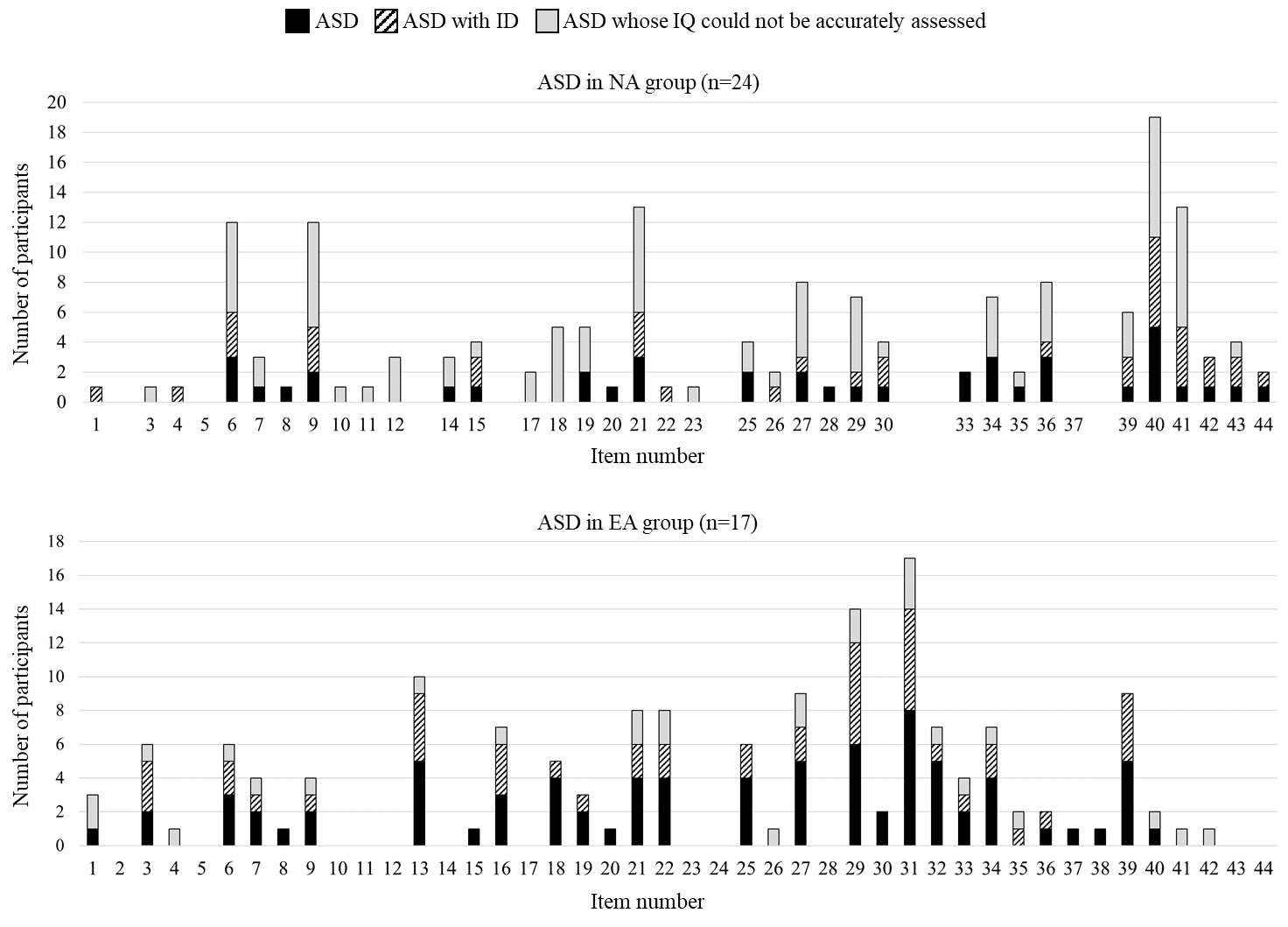


Supplementary Figure S1. The number of participants included in the ASD group who showed posture and movement for each item. ASD; autism spectrum disorder, ID; intellectual disability, IQ; intelligence quotient, NA; neonatal assessment, EA; early infancy assessment
